# E2F7−EZH2 axis regulates PTEN/AKT/mTOR signalling and glioblastoma progression

**DOI:** 10.1038/s41416-020-01032-y

**Published:** 2020-08-20

**Authors:** Rui Yang, Mei Wang, Guanghui Zhang, Yonghua Bao, Yanan Wu, Xiuxiu Li, Wancai Yang, Hongjuan Cui

**Affiliations:** 1 Key Laboratory of Precision Oncology of Shandong Higher Education, Institute of Precision Medicine, Jining Medical University, Jining, China; 2 State Key Laboratory of Silkworm Genome Biology, Southwest University, Chongqing, China; 3 Guizhou Provincial College-based Key Laboratory for Tumor Prevention and Treatment with Distinctive Medicines, Zunyi Medical University, Zunyi, China; 4 Cancer Center, Medical Research Institute, Engineering Research Center for Cancer Biomedical and Translational Medicine, Southwest University, Chongqing, China; 5 Department of Pharmacy, The Second People’s Hospital of Liaocheng, Liaocheng, China; 6 Department of Pathology, University of Illinois at Chicago, Chicago, IL USA

**Keywords:** CNS cancer, Tumour biomarkers

## Abstract

**Background:**

E2F transcription factors are considered to be important drivers of tumour growth. E2F7 is an atypical E2F factor, and its role in glioblastoma remains undefined.

**Methods:**

E2F7 expression was examined in patients by IHC and qRT-PCR. The overall survival probability was determined by statistical analyses. MTT assay, colony formation, cell-cycle assay, cell metastasis and the in vivo model were employed to determine the functional role of E2F7 in glioblastoma. Chromatin immunoprecipitation, luciferase assay and western blot were used to explore the underlying mechanisms.

**Results:**

E2F7 was found to be up-regulated in glioblastoma patients, and high E2F7 expression was associated with poor overall survival in glioblastoma patients. Functional studies showed that E2F7 promoted cell proliferation, cell-cycle progression, cell metastasis and tumorigenicity abilities in vitro and in vivo. E2F7 promoted the transcription of EZH2 by binding to its promoter and increased H3K27me3 level. EZH2 recruited H3K27me3 to the promoter of PTEN and inhibited PTEN expression, and then activated the AKT/mTOR signalling pathway. In addition, restored expression of EZH2 recovered the abilities of cell proliferation and metastasis in E2F7-silencing cells.

**Conclusion:**

Collectively, our findings indicate that E2F7 promotes cell proliferation, cell metastasis and tumorigenesis via EZH2-mediated PTEN/AKT/mTOR pathway in glioblastoma.

## Background

Glioblastoma (GBM) is the most common and aggressive subtype of primary malignant brain tumour in adults.^1,2^ Although multimodality therapeutic scheme, including surgical resection and chemo radiation, has been accepted as a reliable therapy for the treatment of glioblastoma, the prognosis of glioblastoma patients is still very poor, with a median survival of about 15 months, which results in tumour recurrence.^3,4^ The extreme ability of proliferation and invasion is due to anomalous genetic alterations and subsequent signal pathway stimulations.^5^ However, the essential alterations involved in glioblastoma initiation and development remain elusive. Thus, better defining glioblastoma pathogenesis and exploring new anti-cancer agents and therapeutic strategies are urgently needed to improve the quality of life of the glioblastoma patients.

E2F transcription factors are considered to be important drivers of tumour growth, which are involved in various progressions such as cell cycle, angiogenesis and DNA-damage response.^6–9^ E2Fs are classified as typical E2Fs, including E2F1−E2F6 and atypical E2Fs (E2F7 and E2F8).^10^ Typical E2Fs contain a conserved DBD domain (DNA binding) and a DIM domain (protein binding). Typical E2F proteins interact with DP protein to regulate transcription by binding to the promoter of target genes.^11,12^ Unlike typical E2Fs, as an atypical E2F factor, E2F7 contains two individual DNA-binding domains, by which it binds to promoter sites of targets in a DP-independent manner.^13,14^ E2F7 may function as a transcriptional repressor through competitive binding with E2F1 to consensus E2F-binding sites of target genes.^15–17^ In addition, E2F7 has been shown to bind to CDH1 promoter in an E2F1-independent manner; whether this has a functional consequence remains unclear.^18,19^ E2F7 promotes cell proliferation and metastasis in lung adenocarcinoma, liver cancer and head and neck cancer.^20–22^ On the other hand, E2F7 can regulate cancer development by antagonising E2F1-induced proliferation and differentiation in cutaneous squamous cell carcinoma and gallbladder cancer.^7,15^ Thus, the tumour-promoting or -suppressing roles of E2F7 are different in individual cancer types. However, the clinical significance of E2F7 and its role in glioblastoma remains unclear.

In this study, using the online database, patient specimens and in vitro and in vivo models, we studied the genetic alteration, clinical value and biological effects of E2F7 in glioblastoma. In addition, we explored the mechanistic role of E2F7−EZH2 axis in AKT/mTOR activation, and the results suggested that E2F7 promoted glioblastoma progression via EZH2-mediated PTEN/AKT pathway.

## Methods

### Patients’ data

Eighty-four glioma specimens and 12 normal tissues were collected for examination of protein and mRNA levels of E2F7 from the Second People’s Hospital of Liaocheng (Liaocheng, China). A cohort of 84 paraffin-embedded glioma cases was diagnosed from January 2006 to December 2011 at the Second People’s Hospital of Liaocheng. None of the patients had received radiotherapy or chemotherapy before surgery. All samples were anonymous. This project was approved by the Institute Research Ethics Committee of Jining Medical University and the Second People’s Hospital of Liaocheng. The clinical implication of E2F7 was further determined in the Oncomine dataset (https://www.oncomine.org), The Cancer Genome Atlas (TCGA) dataset (http://www.cbioportal.org) and R2: microarray analysis and visualisation platform (http://hgserver1.amc.nl/cgi-bin/r2/main.cgi).

### Cell lines and cell culture

The human glioblastoma cell lines LN229, U251, U118, A172, U87- MG, normal glial cell SVGP12 and human embryonic kidney (HEK) 293FT cells were obtained from ATCC. All the glioblastoma cells and HEK 293FT cells were cultured in Dulbecco’s modified eagle medium (DMEM) (Invitrogen), supplemented with 10% foetal bovine serum (FBS) (Thermo Fisher) and antibiotics. The human normal glial cells SVGP12 was cultured in Minimum Eagle’s medium (MEM) (Invitrogen), supplemented with10% FBS and antibiotics. All cells were maintained in a humidified atmosphere containing 5% CO_2_ at 37 °C.

### Quantitative real-time PCR

The expression levels of various genes were analysed by real-time reverse transcription−polymerase chain reaction (RT−PCR). qRT−PCR was performed according to our previous study.^23^ Primers are shown in Supplementary Table S1. All samples were analysed using a Bio-Rad real-time analyser (Bio-Rad Laboratories), and the results were normalised to the glyceraldehyde-3-phosphate dehydrogenase (GAPDH) expression. Each experiment was repeated three independent times.

### Vector construction, transfection and infection

The hairpin oligonucleotides were synthesised in Beijing Genomics Institute (BGI, Beijing, China) and cloned into the pLKO.1 lentivirus vector. Primer sequences are listed in Supplementary Table S1. The full-length cDNA of human E2F7 and EZH2 gene was generated by PCR and verified by DNA sequencing. pCMV plasmid is the vector for the expression constructs. These were obtained from Youbio (Changsha, China). EZH2 promoter fragment was amplified from human genomic DNA with appropriate sets of primers. Mutations of the consensus E2F7-binding site were synthetic. PCR and synthetic products were cloned into the luciferase vector pGL3-basic. All constructs were verified by sequencing. The E2F7 shRNA, E2F7-overexpression and EZH2-overexpression vectors were transfected into 293FT cells using the Lipofectamine 2000 reagent (Invitrogen, Carlsbad, CA, USA) according to the manufacturer’s protocol. Lentiviruses were collected 48 h later, and were used to infect glioblastoma cells twice, 12 h per infection. The infected cells were screened by treatment for 36 h with puromycin (2 μg/ml), and the surviving cells were frozen and stored in liquid nitrogen for subsequent experiments.

### Cell proliferation and colony-formation assays

As described previously,^23^ cell proliferation was determined by the MTT assay. One-thousand cells were plated onto 96-well microdilution plates, and MTT was added on 5 consecutive days at a final concentration of 5 mg/ml. After incubation at 37 °C and 5% CO_2_ for 4 h, the MTT was removed, and MTT formazan crystals were dissolved in 200 μl of DMSO (dimethyl sulfoxide). Absorbance at 560 nm was determined on an automatised microtiter plate reader (Bio-Rad). For colony-formation assay, 1 × 10^3^ cells were mixed with 0.3% Noble agar in growth medium and plated into six-well plates containing a solidified bottom layer (0.6% Noble agar in growth medium). The colonies were photographed after 14−21 days and recorded.

### BrdU staining

BrdU immunofluorescent staining was performed according to our previous study,^24^ cells were grown on coverslips, and incubated with 10 μg/ml BrdU (Sigma) for 30 min, then washed with phosphate-buffered saline (PBS) and fixed in 4% paraformaldehyde (PFA) for 20 min. Subsequently, cells were pre-treated with 1 mol/l HCl, and blocked with 10% goat serum for 1 h, followed by a monoclonal rat primary antibody against BrdU (1:200, ab6326, Abcam, Cambridge, MA, USA) for 1 h and Alexa FluorR® 594 goat anti-rat IgG secondary antibody (H + L, Invitrogen). DAPI (4',6-diamidino-2-phenylindole) (300 nM) was used for nuclear staining; the percentage of BrdU was calculated at least from ten microscopic fields (Nikon 80i, Nikon Corporation, Tokyo, Japan).

### Cell-cycle assay

As described previously,^24^ 1 × 10^6^ cells were harvested and washed twice with cold PBS, followed by fixation with ice-cold 70% ethanol overnight at 4 °C. After washing twice with PBS, the cells were incubated with propidium iodide (PI) (BD Biosciences) and RNaseA for 30 min at room temperature. The cells were then analysed using a FACS C6 (BD Biosciences) with CellQuest software.

### Cell migration and invasion assays

As described previously,^25^ migration and invasion were examined using a transwell chamber (Millipore). For the migration assay, 1 × 10^5^ transfected cells were plated into the upper chamber, and cultured in DMEM, while DMEM with 10% FBS was added to the lower chamber. After 16 h of incubation at 37 °C, cells remaining on the upper surface of the membrane were removed, and the membrane was stained with 20% methanol and 0.5% crystal violet. Cell images were obtained using an inverted microscope (Olympus). For invasion assays, the upper chamber was precoated with Matrigel (BD Biosciences).

### Western blot

As described previously,^25^ whole-cell lysates were prepared after incubation with RIPA buffer (Sigma) on ice for 10 min. Samples were heated at 95 °C for 5 min and subjected for 10 min to centrifugation at 10,000 × *g*. Cell lysates were separated by 10% SDS-PAGE and were transferred to a polyvinylidene difluoride membrane. SDS-PAGE gels were calibrated using Magic Mark XP Western Standard (Invitrogen). SDS-PAGE was performed, and proteins were detected using their respective antibodies. Bound antibodies were visualised by chemiluminescence using the ECL Prime Western blotting (WB) detection system (GE Healthcare), and luminescent images were analysed with a Lumino Imager (LAS-4000 mini, Fuji Film Inc.). Each experiment was repeated at least three times. The primary antibodies are as follows: E2F7 (Abcam), cyclin D1, cyclin E, CDK2 and CDK4 (Cell Signaling Technology), N-cadherin, E-cadherin, Fibronectin, Vimentin and MMP9 (Cell Signaling Technology), β-catenin (Abcam), EZH2 and PTEN (Abcam), H3K27me3 (Cell Signaling Technology), AKT, p-AKT at Ser473, mTOR and p-mTOR at Ser2448 (Cell Signaling Technology) and GAPDH and β-actin (Abcam).

### Chromatin immunoprecipitation (ChIP) assay

Chromatin was isolated from 20 × 10^6^ LN229, LN229/shCtrl, LN2229/shE2F7, U251/shCtrl and U251/shE2F7 cells. As described previously,^23^ chromatin was crosslinked with formaldehyde (1%), disrupted and immunoprecipitated with magnetic protein G dynabeads (Dynabeads, Life Technologies) using 2 μg of the specific antibodies anti-E2F7, anti-EZH2 and anti-H3K27me3. Immunoprecipitated chromatin was unbound and reverse cross-linked using elution buffer (NaHCO_3_ 100 mM, SDS 1%) at 65 °C. The amount of immunoprecipitated DNA for each specific antibody was quantified in triplicate by quantitative PCR. Values of retrieved DNA were related to the amount of input DNA to quantify the efficiency of the immunoprecipitation. Negative controls included the same samples incubated with rabbit IgG as the primary antibody. Primer sequences are listed in Supplementary Table S1.

### Luciferase assay

As described previously,^23^ cells were transfected with shCtrl or shE2F7 together with the EZH2 reporter or pGL3-basic luciferase reporter and Renilla luciferase plasmid using Lipofectamine 2000. The transfected cells were cultured for 36 h. Cells were lysed and assayed with Dual Luciferase Assay (Promega). Each experiment was repeated three independent times.

### Animal studies

Animal experiments were performed in compliance with the guidelines of the Institute for Laboratory Animal Research, Jining Medical University. Animal welfare and experimental procedures were carried out in accordance with the *Guide for the Care and Use of Laboratory Animals* (Ministry of Science and Technology of China, 2006). Four-week-old male nude mice (BALA/c, Beijing Laboratory Animal Research Center) were purchased and housed in the SPF room to acclimate for a week. Subcuticular injections were performed following a previous protocol with minor modifications.^26^ Briefly, these mice were divided into three groups (4/group). Mice were anaesthetised via intraperitoneal injections of pentobarbital sodium (1%, 0.01 ml/g^1^). Animals were continuously monitored based on neurologic stimulation of the tail and respiration rate. LN229-vector (1 × 10^6^ cells in 100 μl of PBS), LN229-shE2F7 and LN229-shE2F7/EZH2 cells were inoculated subcutaneously into the nude mice on March 2, 2020. The tumour size was measured using a Vernier calliper every 5 days, and the volume was calculated with the following formula: *V* = (length × width^2^)/2. At the termination of the experiment, the tumour mass was harvested, weighed and stored for immunostaining or protein extract.

Before the tumours were collected, the previously described system was used to introduce nasal anaesthesia (isoflurane) into mice to reduce their pain. Then, the mice were killed by cervical dislocation. The bodies of mice were collected and frozen at −20 °C and then transferred to Nuoau Biotech Inc. (Jining, China) to incinerate. All experiments were performed on a sterile workbench of an SPF room at Jining Medical University.

### Immunohistochemistry staining

As described previously,^25^ paraffin-embedded tumour tissues were sectioned at 5 μm, deparaffinised and rehydrated. For antigen retrieval, sections were treated for 20 min at 95 °C in 10 mmol/l citrate buffer (pH 6.0) in a laboratory microwave oven and subsequently washed in PBS. For immunohistochemistry, after quenching of endogenous peroxidase activity and blocking with normal goat serum, sections were incubated sequentially with E2F7, PTEN, p-AKT and p-mTOR primary antibodies, biotinylated goat anti-rabbit IgG and the ABC reagent (Vector Laboratories). The immunostaining was visualised with 3,3′-diaminobenzidine (Sigma). Sections were then counterstained with haematoxylin before being examined using a light microscope.

### Statistical analysis

All observations were confirmed by at least three independent experiments. Quantitative data are expressed as the mean ± standard deviation. Two-tailed Student’s *t* test was performed for paired samples. Kaplan–Meier analyses were used for survival analysis. *P* < 0.05 was considered statistically significant.

## Results

### E2F7 is highly expressed and correlated with poor prognosis in glioblastoma

**Fig. 1:**
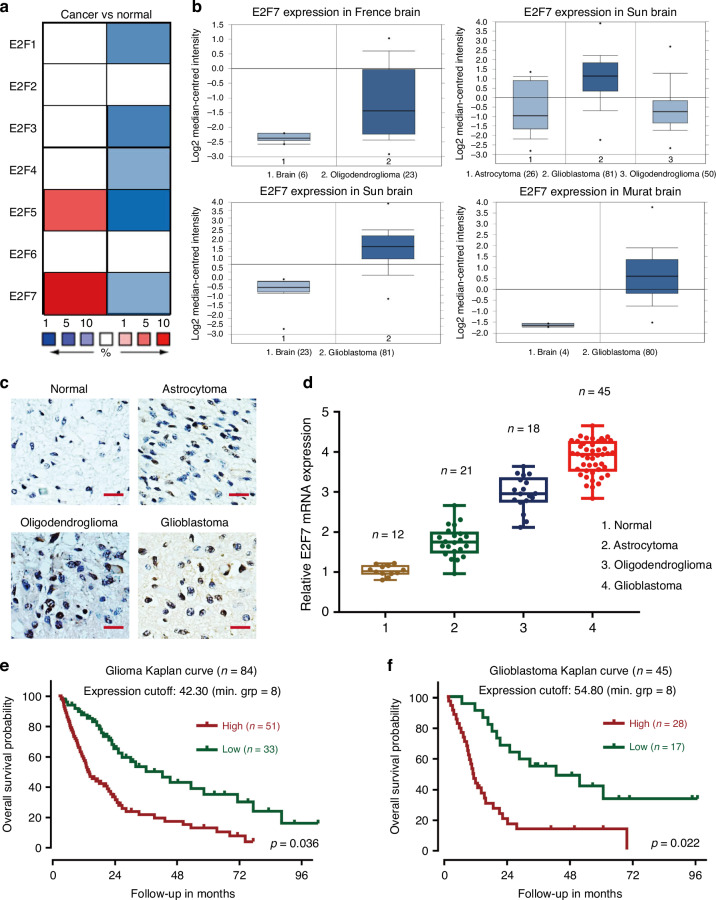
E2F7 expression is increased and correlated with poor outcomes. **a**, **b** Studies from the Oncomine datasets presented the increase in E2F7 mRNA in glioblastoma samples. **c** The expression of E2F7 in normal, astrocytoma, oligodendroglioma and glioblastoma tissues was detected by IHC. **d** The mRNA expression of E2F7 in normal, astrocytoma, oligodendroglioma and glioblastoma tissues was determined by qRT−PCR. **e** The clinical significance of E2F7 expression in glioma overall survivals was evaluated by Kaplan−Meier survival analyses. **f** The clinical significance of E2F7 expression in glioblastoma overall survivals was evaluated by Kaplan−Meier survival analyses.

We first examined the expression of E2F factors in glioma; the result showed that E2F7 was the most significantly highly expressed among the family members in the Oncomine database (Fig. 1a). In addition, based on different brain studies in the Oncomine dataset, E2F7 was frequently up-regulated in glioblastoma (high-grade glioma), compared with normal tissues and oligodendroglioma (low-grade glioma) (Fig. 1b). This result was validated by the TCGA data (Supplementary Fig. S1A). The expression of E2F7 was further detected in fresh glioma tissues by IHC and qRT−PCR; we found that E2F7 was also abnormally amplified in fresh glioblastoma tissues (Fig. 1c, d). We also examined the expression of E2F7 in glioblastoma cell lines; E2F7 expression level was higher in glioblastoma cell lines than in normal glial cell SVGP12 (Supplementary Fig. S2A, B).

We next explored the prognostic implication of E2F7 in glioma and glioblastoma. In the local cohort, glioma patients with high E2F7 expression survived for a shorter time than those with low E2F7 expression (Fig. 1e). This effect was more evident in glioblastoma patients. The median survival time in the high and low E2F7 groups was 11.3 and 18.5 months, respectively (Fig. 1f). The data from the R2 French dataset confirmed the above results (Supplementary Fig. S1B). All the results suggest that E2F7 serves as a promising marker for prognosis of glioblastoma patients.

### E2F7 promotes cell proliferation in glioblastoma

**Fig. 2:**
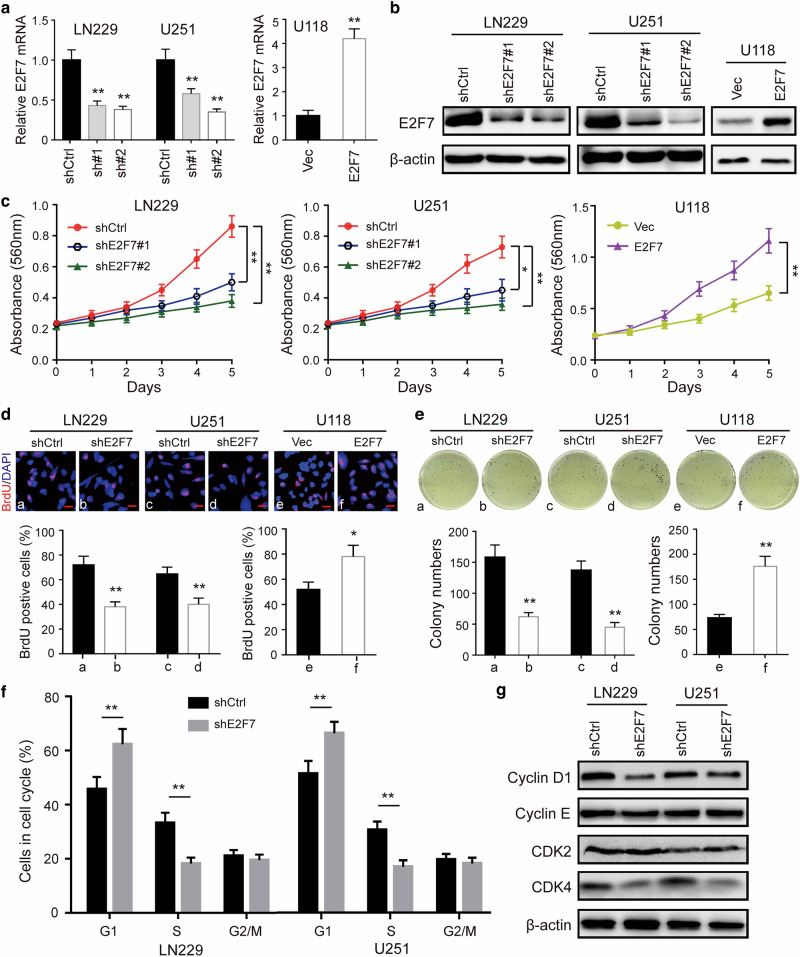
E2F7 is important for cell proliferation in glioblastoma. **a** The mRNA levels of E2F7 in glioblastoma cells transduced with E2F7 shRNA clones or E2F7-overexpression vectors were determined by qRT-PCR. **b** The protein expression of E2F7 in the stable cell lines was examined by western blot. **c** The effect of E2F7 on the proliferation of glioblastoma cells was shown by MTT assays. **d** Image and quantification of glioblastoma cells positive for BrdU staining were shown. **e** Colony formation in soft agar was performed to validate the impact of E2F7 on cell growth. **f** The cell cycle analyses were performed in cells with shE2F7 and E2F7 overexpression. The percentage of cells in the G1 phase was indicated. **g** The protein expression of cyclin D1, cyclin E, CDK2 and CDK4 was examined by western blot. All data are shown as the mean ± SD, *p < 0.05, **p < 0.01.

To investigate the biological function of E2F7 in glioblastoma, LN229 and U251 cells were stably transfected with E2F7 shRNAs, and U118 cells were transfected with E2F7-overexpression vector. E2F7 expression levels in shE2F7- and E2F7-overexpressing cells were examined by qRT-PCR and western blot (Fig. 2a, b). Next, we examined the proliferation kinetics of glioblastoma cells by MTT assays. The results showed that knockdown of E2F7 significantly inhibited cell proliferation, while overexpression of E2F7 induced a significant increase in cell proliferation (Fig. 2c). This result was confirmed by BrdU staining, in which shE2F7 induced a reduction and E2F7 overexpression induced an increment in DNA synthesis (Fig. 2d). Soft-agar assays revealed that the colonies were smaller and lesser in shE2F7 cells, while overexpression of E2F7 promoted colony formation in agar (Fig. 2e).

Since cell-cycle progression is a major regulator of cell proliferation, we analysed cell-cycle progression to determine whether E2F7 promoted proliferation through accelerating the cell-cycle process. Indeed, after E2F7 knockdown, the number of cells was accumulated in the G1 phase. Inversely, overexpression of E2F7 promoted cell-cycle progression in glioblastoma (Fig. 2f). Further evidence was obtained by detecting the expression of cyclins and CDKs by immunoblotting. We found that the expression of cyclin D1 and CDK4 was decreased after E2F7 knockdown, while overexpression of E2F7 increased the levels of cyclin D1 and CDK4 (Fig. 2g). These results indicated that E2F7 promoted proliferation by regulating cell-cycle progression in glioblastoma.

### E2F7 facilitates migration and invasion of glioblastoma cells

**Fig. 3:**
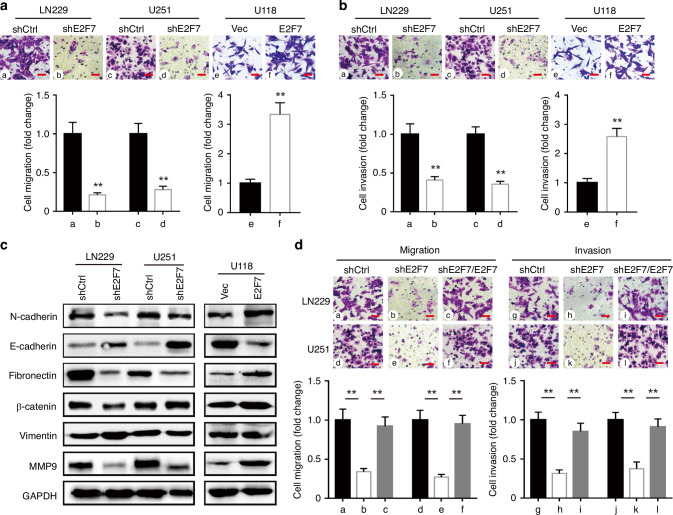
E2F7 promotes cell migration and invasion in glioblastoma. **a** Cells with E2F7 overexpression or silencing were subjected to transwell assays. The migrated cells were stained with 0.05% crystal violet and counted. The representative images and the fold change of cell migration were presented. **b** Invasion assays were performed to determine the effect of E2F7 on cell invasion. **c** Proteins extracted from stable cell lines were subjected to western blot to detect the expressions of EMT-related markers, such as N-cadherin and MMP2. **d** The expression of E2F7 was restored in E2F7-knockdown cells. The representative images and the fold change of cell migration and invasion were presented. All data are shown as the mean ± SD, **p < 0.01.

High invasiveness is an important cause of difficult treatment of glioblastoma; we performed transwell assays to determine whether E2F7 is essential for cell migration and invasion in vitro. The results revealed that E2F7 depletion reduced the migration ability of LN229 and U251 cells, whereas E2F7 overexpression increased the migrated cells in U118 cells (Fig. 3a). In addition, wound-healing assay showed that the migratory ability of glioblastoma cells with E2F7 knocked down was significantly lower than that of the control cells (Supplementary Fig. S3). Invasion assays showed that E2F7 was essential for cell invasion in glioblastoma cells (Fig. 3b). Western blot showed that N-cadherin, fibronectin and MMP9 were down-regulated after E2F7 knockdown, whereas the expression of E-cadherin was increased in E2F7-knockdown cells. In contrast, overexpression of E2F7 induced the inverse effects in the regulation of those proteins (Fig. 3c). In addition, restoration of E2F7 in shE2F7 cells facilitates their abilities of migration and invasion (Fig. 3d). These results demonstrated that E2F7 might promote migration and invasion of glioblastoma cells via triggering EMT process.

### EZH2 is a downstream gene of E2F7

**Fig. 4:**
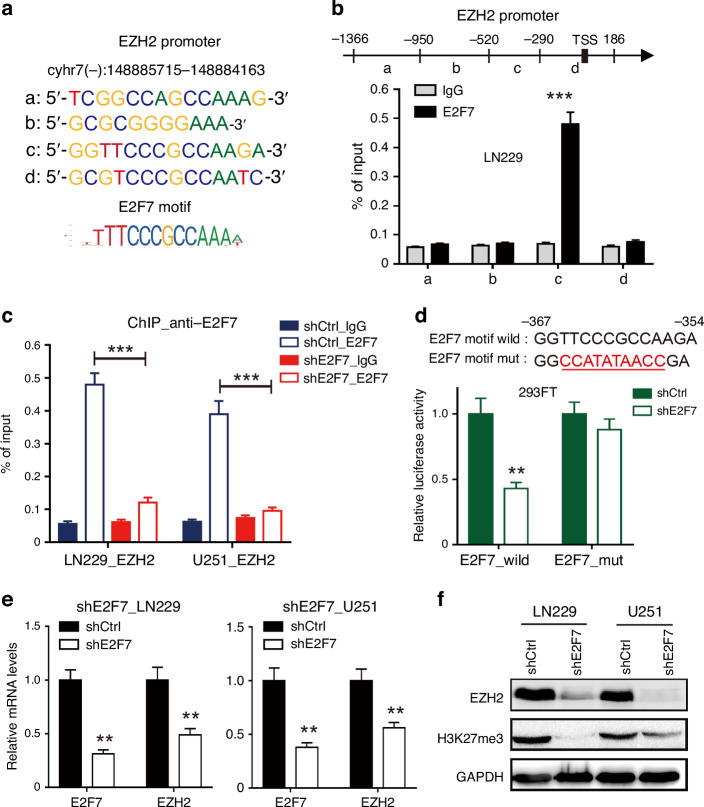
E2F7 is a direct transcriptional activator of EZH2 expression. **a** Predicted sequences of E2F7 motif in *EZH2* promoter by JASPAR. **b** ChIP-qPCR analysis of E2F7 levels at different regions of *EZH2* promoter in LN229 cells. **c** ChIP-qPCR analysis of E2F7 levels at *EZH2* promoter in LN229 and U251 cells expressing shCtrl or shE2F7. **d** Luciferase promoter/reporter constructs were created containing the truncated wild *EZH2* promoter or E2F7 motif mut; then, the EZH2 promoter/reporter constructs were co-transfected with a shE2F7 or with an empty vector (shCtrl) into HEK293FT cells, and luciferase activity was evaluated 24 h later. **e** The mRNA levels of EZH2 in E2F7-silenced cells were determined by qRT-PCR. **f** The protein expression of EZH2 and H3K27me3 in E2F7-silenced cells was examined by western blot. All data are shown as the mean ± SD, **p < 0.01, ***p < 0.001.

Recent studies have identified that epigenetic changes play a crucial role in the carcinogenesis and progression; among them, histone methylation has attracted more attention. To assess the role of E2F7 in the regulation of histone methyltransferase, we analysed the published dataset GSE105353 from a ChIP-seq study of genome-wide E2F7-binding site.^27^ The result showed a specific association of E2F7 with the *EZH2* promoter, in K562 cells, a human chronic myeloid leukaemia cell line (Supplementary Fig. S4A). We found four binding sites of E2F7 in the *EZH2* promoter based on the JASPAR database (Fig. 4a). To further examine the binding site of E2F7 in *EZH2* promoter, we performed a ChIP-qPCR analysis to locate the E2F7-binding site on *EZH2* promoter in glioblastoma cells. In line with the ChIP-seq result, we found that there were many E2F7 proteins enriched in the *EZH2* promoter region (−520 to −290) (Fig. 4b). In addition, the knockdown of E2F7 significantly inhibited the binding of E2F7 with *EZH2* promoter in glioblastoma cells (Fig. 4c).

To further verify whether E2F7 directly promotes the transcription of EZH2, we constructed different luciferase reporter vectors containing *EZH2* promoter with wild E2F7 motif or mutant E2F7 motif, and then detected the effects of E2F7 on luciferase activity of these constructs by performing luciferase assay. We found that E2F7 silencing significantly inhibited EZH2 promoter activity, but knockdown of E2F7 did not affect the activity of EZH2 promoter/reporter construct with mutant E2F7 motif (Fig. 4d). The qRT-PCR and western blot assay showed that E2F7 knockdown resulted in a significant decrease in the expression levels of EZH2 and H3K27me3 (Fig. 4e, f). According to the TCGA and French datasets, EZH2 was up-regulated in glioblastoma, and high expression of EZH2 was correlated with poor patient prognosis (Supplementary Fig. S4B, C). These results together suggest that E2F7 directly promotes the transcription of EZH2 by binding to its promoter.

### E2F7−EZH2 axis triggers the AKT/mTOR pathway in glioblastoma

**Fig. 5:**
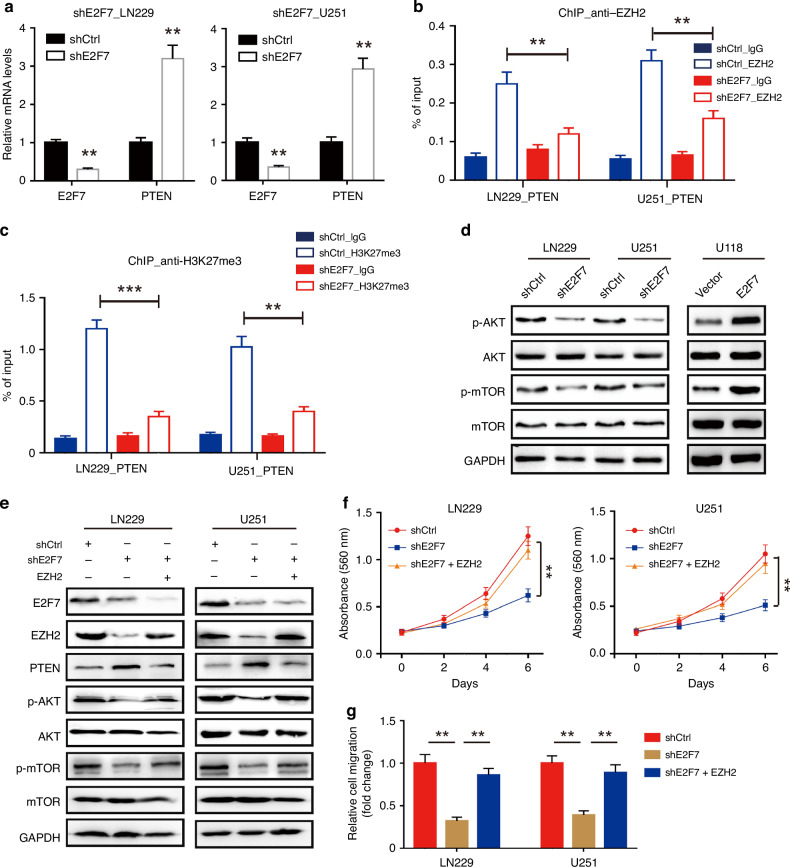
E2F7−EZH2 axis triggers the AKT/mTOR pathway. **a** The mRNA levels of EZH2 and PTEN in E2F7-silenced cells were determined by qRT-PCR. **b** ChIP-qPCR analysis of EZH2 levels at *PTEN* promoter in LN229 and U251 cells expressing shCtrl or shE2F7. **c** ChIP-qPCR analysis of H3K27me3 levels at *PTEN* promoter in LN229 and U251 cells expressing shCtrl or shE2F7. **d** The expression of AKT, p-AKT, mTOR and p-mTOR in glioblastoma cells transduced with E2F7 shRNA clones or E2F7-overexpression vectors was examined by western blot. **e** The expression of E2F7, EZH2, PTEN, AKT, p-AKT, mTOR and p-mTOR in shE2F7 or shE2F7/EZH2 cells was determined by western blot. **f** The effect of EZH2 overexpression on the proliferation of E2F7-knockdown cells was shown by MTT assays. **g** Cells with shE2F7 or shE2F7/EZH2 were subjected to Transwell assays; the fold change of cell migration was presented. All data are shown as the mean ± SD, **p < 0.01, ***p < 0.001.

The tumour suppressor PTEN is a well-known target of EZH2, which is down-regulated in glioblastoma and correlated with poor overall survival of glioblastoma patients (Supplementary Fig. S5). We determined whether E2F7 was a regulator for transcription of PTEN in glioblastoma cells; the results showed that the mRNA level of PTEN was significantly increased after E2F7 knockdown (Fig. 5a). We next detected the binding of EZH2 to the *PTEN* promoter. ChIP-qPCR results revealed that E2F7 knockdown markedly decreased EZH2 enrichment at *PTEN* promoter. Consistently, H3K27me3 levels in *PTEN* promoter were also decreased after E2F7 knockdown (Fig. 5b, c). Thus, E2F7 regulated PTEN transcription in an EZH2-mediated epigenetic repression manner.

Since PTEN is a negative regulator of AKT/mTOR pathway, we further determined whether E2F7/EZH2 axis regulates this pathway. Western blot showed that phosphorylation of AKT at Ser473, mTOR at Ser2448, was decreased in glioblastoma cells with E2F7 knockdown and increased in cells with E2F7 overexpression (Fig. 5d). To examine whether E2F7 promotes PTEN/AKT pathway by regulating EZH2, we restored the expression of EZH2 in E2F7-silencing cells. The results showed that the recovered expression of EZH2 could reverse the inhibition of AKT/mTOR pathway, which was induced by E2F7 knockdown (Fig. 5e). Moreover, restored expression of EZH2 in E2F7-silencing cells accelerated their proliferation and migration (Fig. 5f, g). These results suggested that E2F7−EZH2 axis triggers the AKT/mTOR pathway by inhibiting PTEN in glioblastoma.

### E2F7 promotes tumour growth by regulating PTEN/AKT/mTOR pathway in glioblastoma

**Fig. 6:**
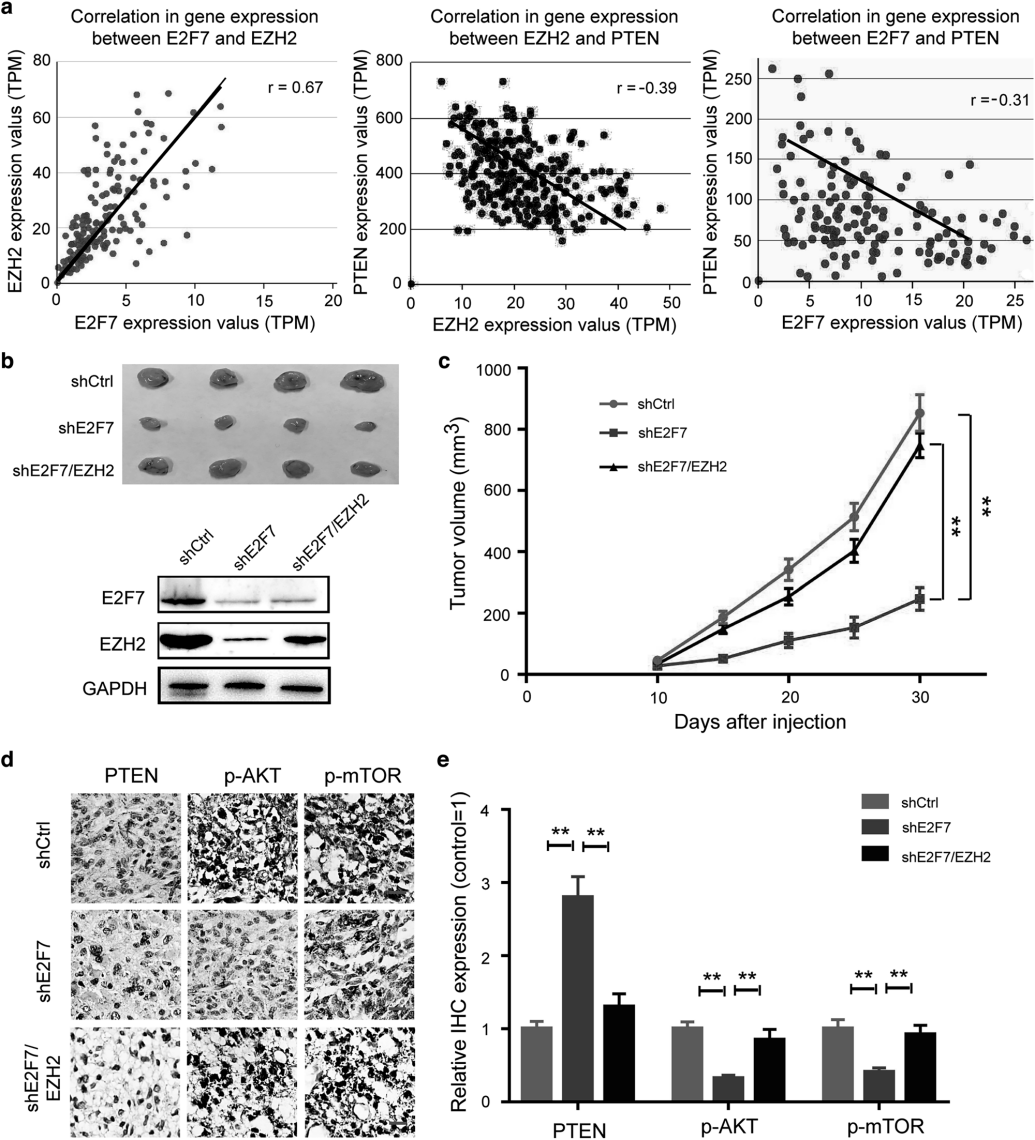
E2F7 promotes tumorigenesis by regulating PTEN/AKT/mTOR pathway in glioblastoma. **a** The correlation of E2F7 expression and EZH2 (left), the correlation of EZH2 expression and PTEN (middle) and the correlation of E2F7 expression and PTEN was determined in the TCGA data. **b** LN229 cells depleted or not depleted of E2F7, and with EZH2 restoration were subcutaneously injected into nude mice; representative dissected tumours and E2F7 expression in tumour lysates are shown. **c** The growth curve of tumours was analysed in indicated times. **d**, **e** The expression of PTEN, p-AKT and p-mTOR in tumour tissues was detected by IHC; the representative images and quantification were shown. All data are shown as the mean ± SD, **p < 0.01.

To evaluate the correlation of E2F7 and EZH2 expression in glioblastoma specimens, we analysed the data from TCGA. We found that E2F7 was positively correlated with EZH2, whereas E2F7 was negatively correlated with PTEN expression in clinical specimens (Fig. 6a). To further investigate the role of E2F7 and EZH2 in tumorigenesis, we injected LN229-shCtrl, LN229-shE2F7 and LN229-shE2F7/EZH2 cells subcutaneously into the nude mice. In the subcutaneous tumour model, E2F7 silencing had significantly inhibited tumour growth compared with control groups, and this effect was rescued by the EZH2 overexpression (Fig. 6b, c). Besides, the expressions of p-AKT and p-mTOR in the tumours formed by E2F7-knockdown cells were decreased compared with the control cells, whereas the expression of PTEN was significantly increased. Nevertheless, the restored expression of EZH2 could reactivate AKT/mTOR pathway in the tumours formed by E2F7-silencing cells (Fig. 6d, e). These results suggested that E2F7−EZH2 axis promoted glioblastoma tumorigenesis by partly accelerating the AKT/mTOR signalling pathway.

## Discussion

Glioblastoma is the most common and aggressive subtype of primary malignant brain tumour in adults and has become a severe challenge to human life.^1^ Although glioblastoma has been extensively studied, the underlying molecular mechanisms are still elusive. More useful biomarkers for the prediction of glioblastoma prognosis are urgently demanded. In this study, we showed that E2F7 was highly expressed and correlated with poor prognosis in glioblastoma, whereas E2F7 expression was not correlated with IDH status, sex, chemotherapy and radiotherapy (Supplementary Table S2). Our data suggest that E2F7 serves as a promising marker for prognosis of glioblastoma patients.

E2F transcription factors are involved in cancer progression, such as E2F1 is considered to be an important driver of tumour growth, including gastric cancer, breast cancer and melanoma.^28–30^ Unlike E2F1, as an atypical E2F factor, E2F7 plays different roles in individual cancer types. E2F7 acts as a competitive factor of E2F1, inhibiting transcriptional activation and tumour promotion induced by E2F1.^15^ On the other hand, E2F7 is significantly increased during formation of tumour spheres in LCSCs.^21^ In our study, we found that E2F7 promoted cell proliferation by regulating cell-cycle progression and migration and invasion of glioblastoma cells via triggering EMT process. Our data suggest that E2F7 functions as an oncogene to promote glioblastoma progression and serves as a promising target for anti-glioblastoma treatment.

Recent studies have identified that epigenetic changes play a crucial role in carcinogenesis and progression; among them, histone methylation has attracted more attention.^31,32^ EZH2 is a lysine methyltransferase, which contains a C-terminal catalytic SET domain.^33^ EZH2 is the essential catalytic enzyme for the tri-methylation of histone H3 lysine 27 (H3K27me3) in mammalian cells.^34^ Accumulated evidence suggests that EZH2 and its product H3K27me3 are correlated with poor outcomes in various human malignancies.^35,36^ EZH2-dependent abnormal transcriptome is involved in carcinogenic process, including EMT,^37,38^ cell-cycle regulation,^39^ differentiation,^40^ tumour immunity process and cell metabolism.^41–43^ Previous studies have revealed some transcription factors that regulate EZH2 expression, including Ras mutations, MEK-ELK1, NF-κB and hypoxia-induced HIF-1α.^44–46^ Here, we identified an E2F7 motif in the EZH2 promoter based on ChIP-seq study. Further studies demonstrated that E2F7 directly promoted the transcription of EZH2 by binding to its promoter site (TTCCCGCCAA). In addition, based on the analysis of glioblastoma patient specimens, we found that EZH2 expression was positively correlated with E2F7, and high expression of EZH2 was correlated with poor patient prognosis. Unlike typical E2Fs, E2F7 lacks the transactivation domain, which activates gene transcription. We speculate that E2F7 is involved in the transcriptional regulation of EZH2 as a coactivator. We detected E2F1 enrichment in E2F7-binding site of EZH2, but there is no E2F1 protein found in this site (Supplementary Fig. S6). Therefore, further studies are needed to identify other activators that work with E2F7 in regulating the transcription of EZH2.

The tumour suppressor PTEN has been proved to be a target gene of EZH2, by which EZH2 could recruit H3K27me3 to the promoter of PTEN and inhibited the transcription of PTEN.^47^ PTEN is an inhibitor of PI3K/AKT pathway, which is frequently down-regulated or loss-of-functionally mutated in a variety of cancers. PTEN suppresses glioblastoma oncogenesis through increasing the enrichment of H3.3 to chromatin to repress gene expression.^48^ PTEN deletion promotes glioblastoma progression, whereas the inhibition of PI3K/AKT pathway by LY294002 attenuates the outcome. In addition, a combination of adenoviral-mediated PTEN and PI3K inhibitor inhibits the malignant growth of glioblastoma cells.^49^ In our study, knockdown of E2F7 increased the expression of PTEN by decreasing the recruitment of EZH2 and H3K27me3 to the promoter of PTEN in glioblastoma cells. Moreover, silencing PTEN attenuated the potency of E2F7 knockdown in glioblastoma cells. Therefore, PTEN likely functions as a bridge between E2F7−EZH2 axis and glioblastoma growth. The prognosis of glioblastoma patients with activated PI3K/AKT/mTOR pathway is poor than patients without oncogenic activation of this pathway. In the present study, E2F7 triggered the AKT/mTOR pathway by inhibiting PTEN in glioblastoma. Restored expression of EZH2 could reactivate AKT/MTOR pathway in the tumours formed by inhibiting PTEN. These results suggested that E2F7−EZH2 axis promoted glioblastoma tumorigenesis by partly regulating PTEN/AKT/mTOR signalling pathway.

## Conclusions

In summary, we showed that up-regulation of E2F7 in glioblastoma was closely correlated with poor prognosis, highlighting the importance of E2F7 in promoting cell proliferation, cell metastasis and tumorigenesis in glioblastoma. We also demonstrated, for the first time, that E2F7 is involved in PTEN/AKT/mTOR pathway through regulating EZH2 in glioblastoma. The newly identified E2F7/EZH2/PTEN axis is a promising target for clinical intervention of glioblastoma.

## Supplementary information

Supplementary materials

## Data Availability

Data and material shall be available from the corresponding authors.
