# Supplementary material for: E2F7−EZH2 axis regulates PTEN/AKT/mTOR signalling and glioblastoma progression
Source: Br J Cancer. 2020 Aug 20;123(9):1445–55. doi: 10.1038/s41416-020-01032-y (PMC7591888; doi:10.1038/s41416-020-01032-y)
Supplement: Supplementary file 1 — Supplementary materials [file 41416_2020_1032_MOESM1_ESM.docx]

**Supplementary Figure S1**


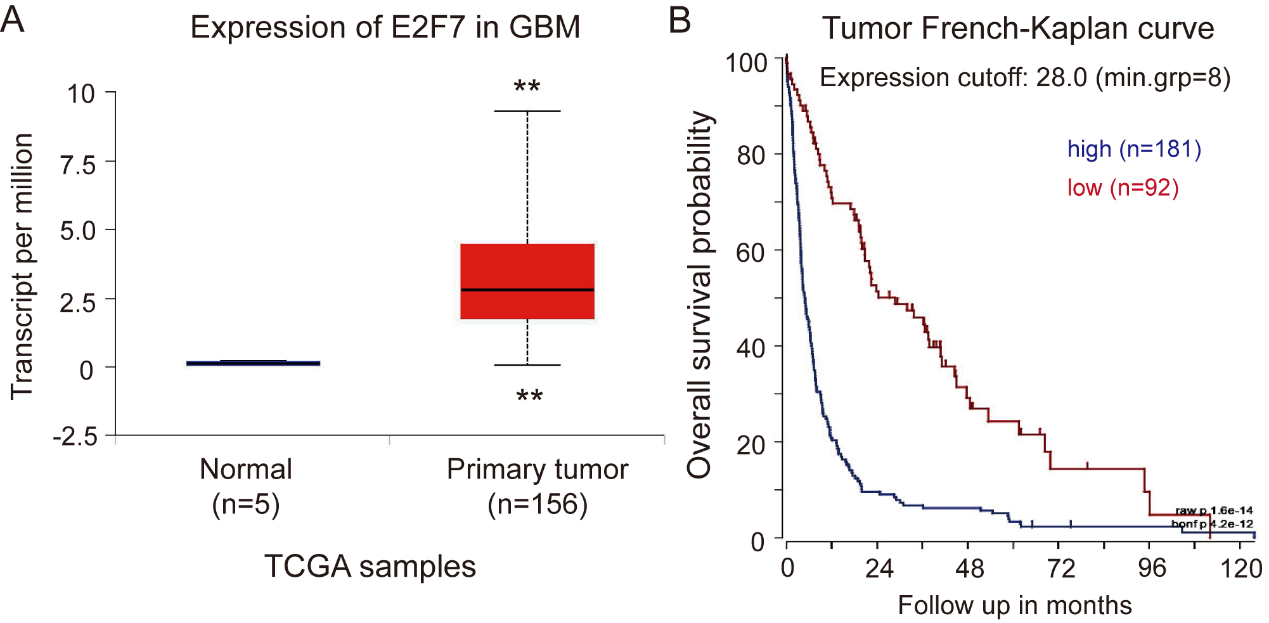


**Supplementary Figure S2**


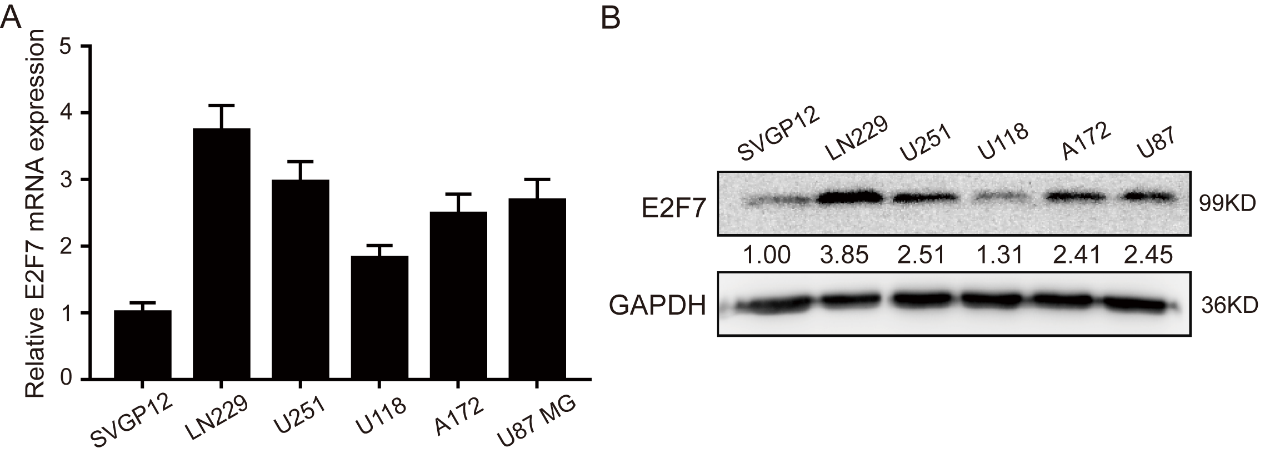


**Supplementary Figure S3**


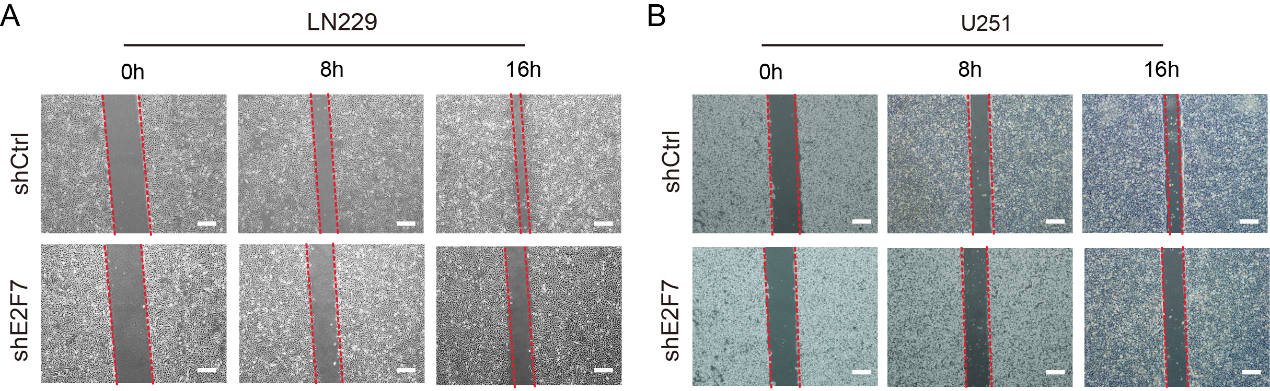


**Supplementary Figure S4**


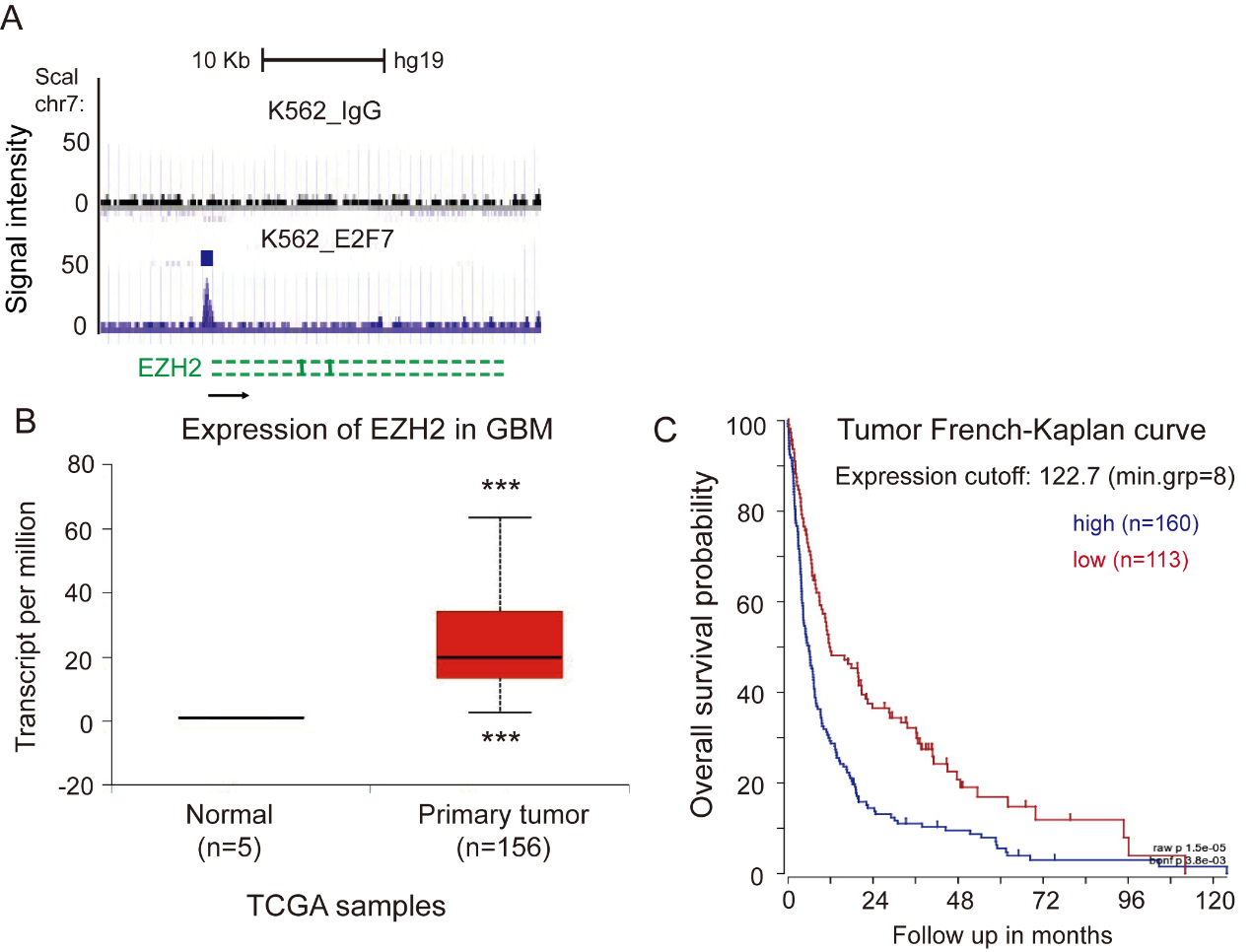


**Supplementary Figure S5**


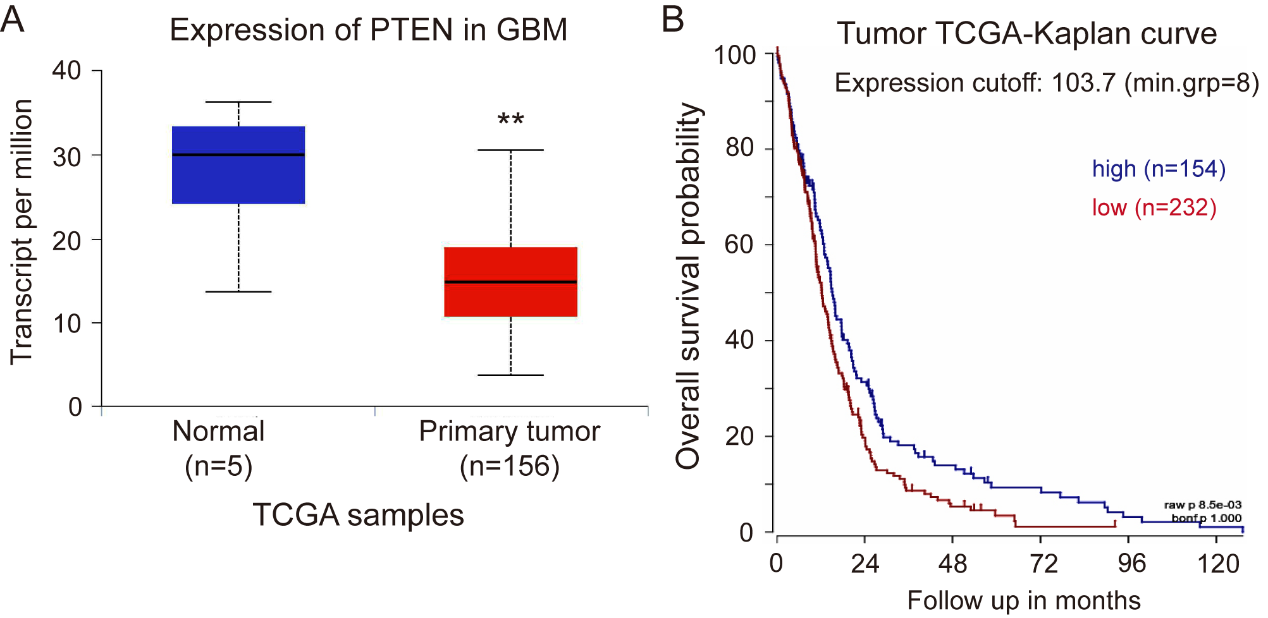


**Supplementary Figure S6**


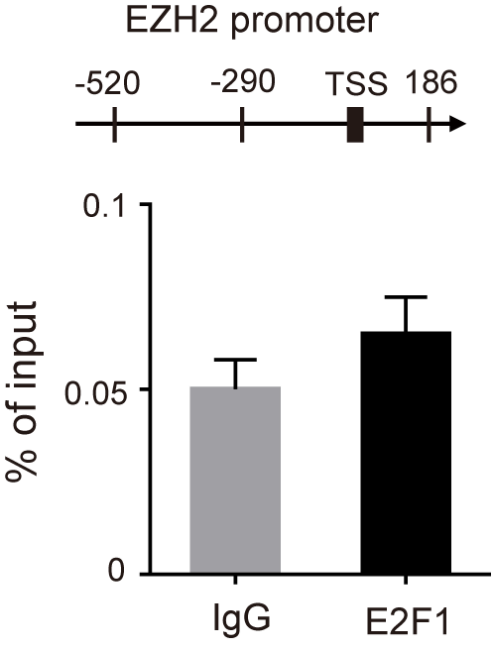


**Supplementary Method**

**Wound-healing assay**

The mean numbers of cells were calculated from at least six randomly chosen microscopic images. For the cell wound-healing assay, cells were cultured in a six-well plate, and wounds were made with a 10-µl pipette tip. Finally, the healing process of cells was observed by microscopy.

**Supplementary Figure Legends**

**Figure S1. E2F7 is up-regulated and correlated with poor outcomes in GBM. A.** The expression of E2F7 in normal and GBM samples was analyzed in TCGA dataset. **B.** Kaplan–Meier analysis of overall survival for the Frence database with the log rank test *P* value indicated. All data were shown as the mean ± SD, **p < 0.01.

**Figure S2. The expression of E2F7 in GBM cell lines was detected. A.** The mRNA expression of E2F7 in normal glio cell SCGP12 and GBM cell lines was determined by qRT-PCR. **B.** The protein level of E2F7 in in normal glio cell SCGP12 and GBM cell lines was examined by western blot.

**Figure S3. Knockdown of E2F7 inhibites cell migration.** Wound-healing assay was performed with E2F7-knockdown LN229 and U251 cells.

**Figure S4. EZH2 is up-regulated and correlated with poor outcomes in GBM. A.** ChIP-seq tag profiles for E2F7 levels at the EZH2 promoter in A562 cells. The ChIP-seq dataset GSE105353 was used for analysis. **B.** The expression of EZH2 in normal and GBM samples was analyzed in TCGA dataset. **C.** Kaplan–Meier analysis of overall survival for the Frence database with the log rank test *P* value indicated. All data were shown as the mean ± SD, ***p < 0.001.

**Figure S5. PTEN is down-regulated in GBM. A.** The expression of PTEN in normal and GBM samples was analyzed in TCGA dataset. **B.** Kaplan–Meier analysis of overall survival for the Frence database with the log rank test *P* value indicated. All data were shown as the mean ± SD, **p < 0.01.

**Figure S6. E2F1 does not bind to *EZH2* promoter.** ChIP-qPCR analysis of E2F1 levels at *EZH2* promoter in GBM cells.

**Table S1** Primers and shRNA sequences used in this study

Primers for qRT-PCR

| Primer name 5′-3 | 5′-3′ |
| --- | --- |
| E2F7-F  E2F7-R  EZH2-F | AAAGGGACTATTCCGACCCAT  ACTTGGATAGCGAGCTAGAAACT  AATCAGAGTACATGCGACTGAGA |
| EZH2-R | GCTGTATCCTTCGCTGTTTCC |
| PTEN-F | TTTGAAGACCATAACCCACCAC |
| PTEN-R | ATTACACCAGTTCGTCCCTTTC |
| GAPDH-F | AACGGATTTGGTCGTATTGGG |
| GAPDH-R | CCTGGAAGATGGTGATGGGAT |

Primers for ChIP assay

| Primer name 5′-3 | 5′-3′ |
| --- | --- |
| EZH2-a-F  EZH2-a-F  EZH2-b-F  EZH2-b-F  EZH2-c-F  EZH2-c-F  EZH2-d-F  EZH2-d-F  PTEN -F (754~954) | CAGTGCTCATCTTGATAAAGGATTT  CCCACCGCTGGACTTTG  GCAGCAGCAGACATAATCAAACA  CTGGCCGCTCCTAGTGGT  TAAAACCGTTACCACCCCCG  CAGGAAGGCGGTGTGCAG  AGAGGCGCCGTGTGTTC  GGACCGAGCGCCAAACG  GGAGGCAGCCGTTCGGAGGATTATT |
| PTEN -R | GGAAATGGCTCTGGACTTGGCGGTA |
| GAPDH-F | GGTAGGGAGTTCGAGACCAG |
| GAPDH-R | TCAACGCAGTTCAGTTAGGC |

Primers for shRNA

| Target Sequence | 5′-3′ |
| --- | --- |
| shE2F7#1-F | CCGGGCAGTCTCCTGCAGGATTAAACTCGAGTTTAATCCTGCAGGAGACTGCTTTTTG |
| shE2F7#1-R | AATTCAAAAAGCAGTCTCCTGCAGGATTAAACTCGAGTTTAATCCTGCAGGAGACTGC |
| shE2F7#2-F | CCGGCTGCGCTAGACTTGGATATTTCTCGAGAAATATCCAAGTCTAGCGCAGTTTTTG |
| shE2F7#2-R | AATTCAAAAACTGCGCTAGACTTGGATATTTCTCGAGAAATATCCAAGTCTAGCGCAG |

**Table S2** Relationship between E2F7 expression and clinicopathologic parameters of 45 glioblastoma patients

| Variable | E2F7 expression | | |
| --- | --- | --- | --- |
|  | Low (n=17) | High (n=28) | P-value |
| Age(years) |  |  | 0.0035 |
| <45 | 11 | 10 |  |
| ≥45 | 6 | 18 |  |
| IDH status |  |  | 0.2321 |
| Mutant | 7 | 11 |  |
| Not mutant | 10 | 17 |  |
| Sex |  |  | 0.1571 |
| Male | 9 | 15 |  |
| Female | 8 | 13 |  |
| Chemotherapy |  |  | 0.3452 |
| Yes | 9 | 19 |  |
| No | 6 | 9 |  |
| NA | 2 | 0 |  |
| Radiotherapy |  |  | 0.0857 |
| Yes | 12 | 22 |  |
| No | 4 | 5 |  |
| NA | 1 | 1 |  |
